# Nfinder: automatic inference of cell neighborhood in 2D and 3D using nuclear markers

**DOI:** 10.1186/s12859-023-05284-2

**Published:** 2023-06-03

**Authors:** Bruno Moretti, Santiago N. Rodriguez Alvarez, Hernán E. Grecco

**Affiliations:** 1 Universidad de Buenos Aires, Facultad de Ciencias Exactas y Naturales, Departamento de Física, Buenos Aires, Argentina; 2 CONICET - Universidad de Buenos Aires, Instituto de Física de Buenos Aires (IFIBA), Buenos Aires, Argentina; 3 Present Address: Department of Molecular and Cell Biology, University of California at Berkeley, Berkeley, USA; 4 Present Address: Institute of Physics, Swiss Federal Institute of Technology Lausanne (EPFL), Lausanne, Switzerland; 5 Present Address: Chan Zuckerberg Biohub—San Francisco, San Francisco, USA

**Keywords:** Microscopy, Image analysis, Cell–cell interactions, Tissues, Neighboring cells, Delaunay triangulation

## Abstract

**Background:**

In tissues and organisms, the coordination of neighboring cells is essential to maintain their properties and functions. Therefore, knowing which cells are adjacent is crucial to understand biological processes that involve physical interactions among them, e.g. cell migration and proliferation. In addition, some signaling pathways, such as Notch or extrinsic apoptosis, are highly dependent on cell–cell communication. While this is straightforward to obtain from membrane images, nuclei labelling is much more ubiquitous for technical reasons. However, there are no automatic and robust methods to find neighboring cells based only on nuclear markers.

**Results:**

In this work, we describe Nfinder, a method to assess the cell’s local neighborhood from images with nuclei labeling. To achieve this goal, we approximate the cell–cell interaction graph by the Delaunay triangulation of nuclei centroids. Then, links are filtered by automatic thresholding in cell–cell distance (pairwise interaction) and the maximum angle that a pair of cells subtends with shared neighbors (non-pairwise interaction). We systematically characterized the detection performance by applying Nfinder to publicly available datasets from *Drosophila melanogaster*, *Tribolium castaneum*, *Arabidopsis thaliana* and *C. elegans*. In each case, the result of the algorithm was compared to a cell neighbor graph generated by manually annotating the original dataset. On average, our method detected 95% of true neighbors, with only 6% of false discoveries. Remarkably, our findings indicate that taking into account non-pairwise interactions might increase the Positive Predictive Value up to + 11.5%.

**Conclusion:**

Nfinder is the first robust and automatic method for estimating neighboring cells in 2D and 3D based only on nuclear markers and without any free parameters. Using this tool, we found that taking non-pairwise interactions into account improves the detection performance significantly. We believe that using our method might improve the effectiveness of other workflows to study cell–cell interactions from microscopy images. Finally, we also provide a reference implementation in Python and an easy-to-use napari plugin.

**Supplementary Information:**

The online version contains supplementary material available at 10.1186/s12859-023-05284-2.

## Background

Understanding the emergence of tissue organization requires quantifying the local and global signaling cues that each cell perceives from the environment. Among these cues, cell–cell interaction is one of the strongest to determine cellular fate during development and maintain homeostasis in adult life. For example, cell–cell contact has an important role during cell migration. A cell in a migrating tissue can change the direction of its movement after direct contact with another cell, in a process that is known as contact inhibition of locomotion (CIL). The number of neighboring cells can alter the outcome of CIL: in a sheet of cells with a free edge, only the cells at the free edge produce lamellipodia, which in turn induces a collective and directed migration of the whole tissue [1]. The number of neighboring cells in a tissue also plays an important role in proliferation. When normal, noncancerous cells are in crowded conditions, they cease proliferation and cell division. This characteristic is lost when cells undergo malignant transformation, resulting in uncontrolled proliferation and tumor formation [2].

At the molecular level, cell–cell interaction is also crucial in a plethora of biological processes. For example, the Notch signaling pathway is highly conserved and plays a key role in cell–cell communication. Since most of this pathway’s ligands are transmembrane proteins, signalling is restricted to neighboring cells [3]. In vertebrate embryonic development, the morphological segments—termed somites—that prefigure the bones and muscles of the adult are formed at the posterior end of the elongating body axis. A highly conserved biological clock consisting of an oscillatory gene regulatory network rhythmically differentiates cells into somites [4]. Here, the Notch signaling pathway plays a key role in synchronizing oscillations between neighboring cells [5]. Disruption of the Notch pathway during development causes severe somite malformation in zebrafish, chick and mouse embryos [6–11].

Thus, knowledge of neighbor relations between cells in tissues is crucial to understanding the emergent collective behaviours that arise from individual cell–cell interactions. Assessing neighborhood in tissues using fluorescence microscopy, i.e. precisely identifying which cells share a border, requires labeling cellular membranes. While this is routinely done in a variety of experiments, it is also many times put aside for technical reasons or convenience. For instance, nuclear markers provide a better signal-to-noise ratio and are spatially well-separated, which simplifies cell counting and identification [12–14]. Due to their brightness, they are also commonly used to perform autofocus routines and track cell movements over time [15–18]. Although it would be ideal to use both nuclei and membrane labelling, this is more expensive, takes a longer acquisition time and adds experimental complexity. For these reasons, nuclei labeling is ubiquitously found in literature both for fixed and live-cell imaging, being DAPI and H2B-FP among the most common.

In this work, we developed Nfinder, a fast, automatic and robust method to find the first neighbors of cells in tissues using only a nuclear marker. To validate our method, we applied it to datasets containing both a nuclear and a membrane marker from a variety of organisms such as *Drosophila melanogaster*, *Tribolium castaneum*, *Arabidopsis thaliana* and *C. elegans*. These datasets were manually analyzed to generate ground truth images, which were in turn used to compare our automatically-found results against. We found that Nfinder is robust and precise for all the test datasets we used, covering 95% of true neighboring relationships with 94% precision, on average.

## Methods

### Delaunay triangulation approximates neighboring cells

**Fig. 1:**
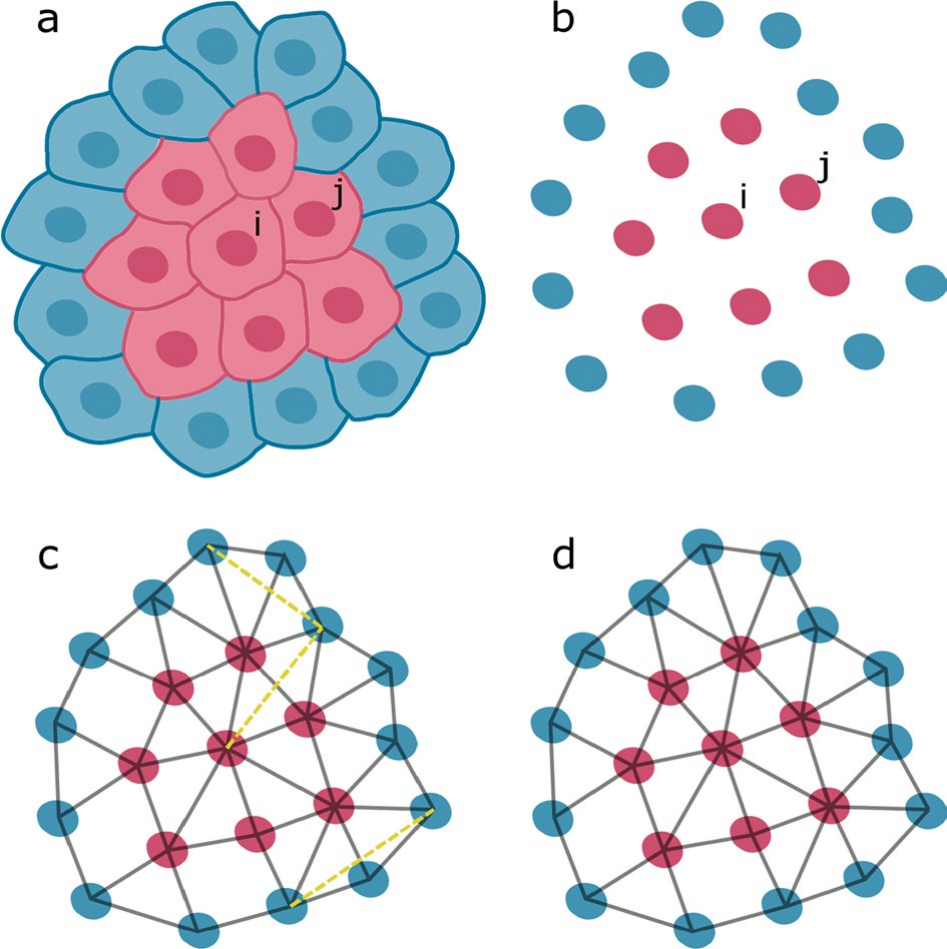
Overview of the proposed method. **a** System of touching cells, showing membranes and nuclei. Cell *i* and its neighbors *j* are highlighted (red). **b** Nuclei corresponding to the cells shown in (**a**). **c** Triangulation of nuclei centroids, including neighboring cells (gray, solid lines) and typical errors (yellow, dashed lines). **d** Final result obtained by filtering the triangulation shown in (**c**)

The goal of our method is to estimate which cells are in contact based only on the positions of nuclei centroids (Fig. 1a, b). Considering this restriction, we define neighboring cells (Fig. 1, red cells) as those which are (1) close together and (2) not interfered by any other cell. This implies that the building blocks of the cell graph are not edges but triangles, because they are the minimal structures that can provide information about the influence of the graph over a given pair of nodes. Consequently, a triangulation of nuclei centroids could be a good approximation of the neighboring cells network (Fig. 1c). In particular, a Delaunay triangulation is outstanding for this task due to its special properties, which result from being the dual of the Voronoi diagram [19]. The latter divides a set of points *S* into touching convex regions, each of which contains only one point in *S*. If two regions are in contact in the Voronoi diagram, they are linked in the Delaunay triangulation. As a result of this, the Delaunay triangulation includes the nearest neighbor graph, which is a necessary (but not sufficient) requirement for any approximation of cell–cell physical interactions. In addition to this, given that Voronoi cells are geometrically stable with respect to small changes of the points in *S*, so the edges of the Delaunay triangulation are [20]. This property ensures that small displacements of cells do not affect the results, distinguishing this triangulation from others. Another essential feature in planar spaces is that the Delaunay triangulation maximizes the minimum angle in any triangle, avoiding sharp structures that would appear if cells with an obstacle in between were assumed to be connected [21].

### Automatic communicability efficiency filter

**Fig. 2:**
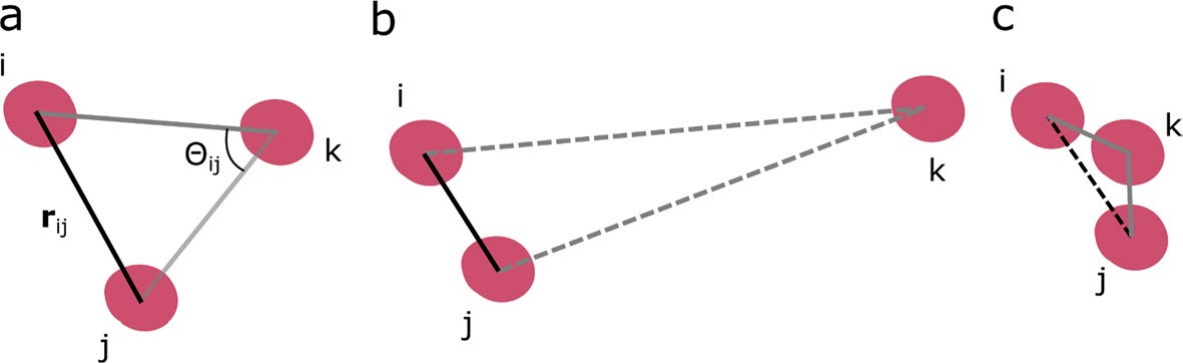
Characterization of the neighboring cells network. **a** Nuclei (red) are connected by solid edges representing cell membrane physical contact. Edge (*i*, *j*) is characterized by nucleus-nucleus distance $r_{ij}$ and the maximum angle subtended with nearby nuclei $\Theta_{ij}$. **b** Small-angle limit, showing *i* and *j* close together while *k* is far away. As a result of this, (*i*, *j*) are neighbors and *k* is isolated from both *i* and *j*, which is indicated by dashed edges. **c** Large-angle limit, in which *k* is potentially blocking the connection between *i* and *j*

By virtue of these attributes, we designed Nfinder, a method to find the first neighbors of cells within tissues using only nuclear markers, based on an implementation of Delaunay triangulations present in the open-source library SciPy [22]. As mentioned before, the Delaunay triangulation is necessary but not sufficient to determine true neighboring relations and, therefore, a filtering procedure is needed (Fig. 1d). To achieve this goal, we characterized each pair of nuclei (*i*, *j*) by their distance $r_{ij}$ and the maximum angle subtended with other nuclei $\Theta_{ij}$ (Fig. 2a). The latter measures the interplay between (*i*, *j*) and its neighborhood, as it informs about how other cells might be blocking the path connecting *i* and *j*.

To understand the role of $\Theta_{ij}$, it is instructive to analyze the possible outcomes for a set of three cells (*i*, *j, k*) in two extreme scenarios. If (*i*, *j*) are close to each other while *k* is very distant,$\Theta_{ij}$ is very small, indicating that (*i*, *j*) cannot be interfered by *k* (Fig. 2b.). On the other hand, when *k* is near to the midpoint of the pair, it will be very unlikely that (*i*, *j*) get in contact, no matter how close they are (Fig. 2c). Here, the relevance of $\Theta_{ij}$ can be appreciated: its value depends on the local spacing between cells, ranging from 0°, when (*i*, *j*) are isolated, to 180°, when there is a neighbor in between. Accordingly, (*i*, *j*) are said to be neighbors if $r_{ij} \le r^{*}$ and $\Theta_{ij} \le \Theta^{*}$, where $r^{*}$ and $\Theta^{*}$ are the maximum values of distance and angle determined by the preference of the biological system towards a particular spatial organization. To estimate these parameters, and based on a previous work that introduced the concept of communicability angle between a pair of nodes in a graph [23], we developed a new statistic that measures cell–cell communicability efficiency, defined as

1 $$E\left( {r, \Theta } \right) = F\left( {r, \Theta } \right) - L\left( {r, \Theta } \right),$$

where *F* is the fraction of connected pairs and *L* is the communicability efficiency loss given that the thresholds are *r* and $\Theta$. Intuitively, the communicability efficiency is a metric that weighs the actual number of connected cells against the number of cells that would be connected if the spatial distribution of cells were random. Consistently, the maximum communicability efficiency would be achieved by connecting the maximum number of cells at the minimum cost. The proposed critical values $r^{*}$ and $\Theta^{*}$ are those that maximize the communicability efficiency of the cell graph

2 $$\left( {r^{*} , \Theta^{*} } \right) = {\text{arg}}\;{\text{max}}\;E\left( {r, \Theta } \right).$$

When the thresholds are $r_{min}$ and $\Theta_{min}$, there are no connectivity losses ($L = 0$). However, this goes to the detriment of the communicability efficiency, as only one pair would be connected. Conversely, when the thresholds are $r_{max}$ and $\Theta_{max} = 180^{ \circ }$, then $L = 1$ and $F = 1$, leading to null communicability efficiency. For this reason, maximizing the communicability efficiency provides a good thresholding criteria, as it balances out the costs and benefits of increasing the critical values of distance and angle.

It is worth noticing that *F* is simply calculated as the Empirical Cumulative Distribution Function (ECDF) of *r* and $\Theta$, which corresponds to the fraction of pairs with $r_{ij} \le r$ and $\Theta_{ij} \le \Theta$. On the other hand,* L* is not unequivocally determined by *r* and $\Theta$. The only constraint on the loss function is that it must be an increasing function of both *r* and $\Theta$. In this paper, we show that measuring loss as

3 $$L_{mean} \left( {r, \Theta } \right) = \frac{1}{2}\left( {\frac{{r - r_{min} }}{{r_{ma x} - r_{min} }} + \frac{{\theta - \theta_{min} }}{{\theta_{max } - \theta_{min} }}} \right)$$

yields accurate predictions on the neighbor graph. Mathematically, $L_{mean}$ is the average probability of randomly choosing *r* and $\Theta$ as the critical values. Since biological systems are not completely random, not all *r* and $\Theta$ are equally probable. As a result of this, there must be some values $\left( {r^{*} , \Theta^{*} } \right)$ that maximize the communicability efficiency of the cell graph and, therefore, are good estimators of the critical values of distance and angle. Source code of a reference implementation in Python for 2D and 3D datasets as well as a user-friendly napari plugin can be found at https://github.com/santi-rodriguez/nfinder.

### Performance metrics used to validate Nfinder

To validate our method, we applied it to the nuclear channel of datasets containing both nuclear and membrane labelling. For each dataset, the double staining allowed us to manually generate the neighboring cells graph of each dataset. First, we segmented and labelled each nucleus via intensity thresholding. Based on this labelling, we manually created a list of connected nuclei defined as those whose membranes were in contact according to what the membrane channel showed. This list of touching cells was used as a ground truth for the neighboring cells graph and compared to our automatic estimation based on nuclear information only. Edges from the estimated graph that matched the ground truth were labelled as true positives (TP). On the contrary, all pairs connected by the algorithm that were not actual neighbors were recorded as false positives (FP). Finally, false negatives (FN) were assigned to touching cells that the algorithm did not detect, while true negatives (TN) corresponded to those edges that were not connected in the ground truth nor in the estimated cell graph.

The metrics used to quantify the performance of the method were the True Positive Rate (TPR)

4 $$TPR = \frac{TP}{{TP + FN}} .$$

Positive Predictive Value (PPV)

5 $$PPV = \frac{TP}{{TP + FP}}$$

and Jaccard Index (JI)

6 $$JI = \frac{TP}{{TP + FN + FP}}.$$

The TPR measures the coverage of positives, while the PPV indicates the fraction of true neighbors among all predictions. Finally, the JI reports the overlap between the list of computed cell–cell edges and the ground truth. Additionally, in the Additional file 1, we show the F1-score

7 $$F1 = \frac{2TP}{{2TP + FP + FN}}$$

and the Matthews correlation coefficient (MCC)

8 $$MCC = \frac{TP \times TN - FP \times FN}{{\sqrt {\left( {TP + FP} \right) \times \left( {TP + FN} \right) \times \left( {TN + FP} \right) \times \left( {TN + FN} \right)} }} .$$

The F1-score represents the harmonic mean of PPV and TPR, while the MCC is the Pearson correlation coefficient between the ground truth classification and the output of the algorithm.

Remarkably, Nfinder is deterministic and scale, reflection and rotational invariant. Thus, it does not depend on knowing the typical distance between neighboring cells, as this is inferred from the image itself. In the next section, we show that neighboring cells can be precisely detected with this method in very diverse tissues.

## Results

### Delaunay triangulation-based method can be applied to diverse tissues

**Fig. 3:**
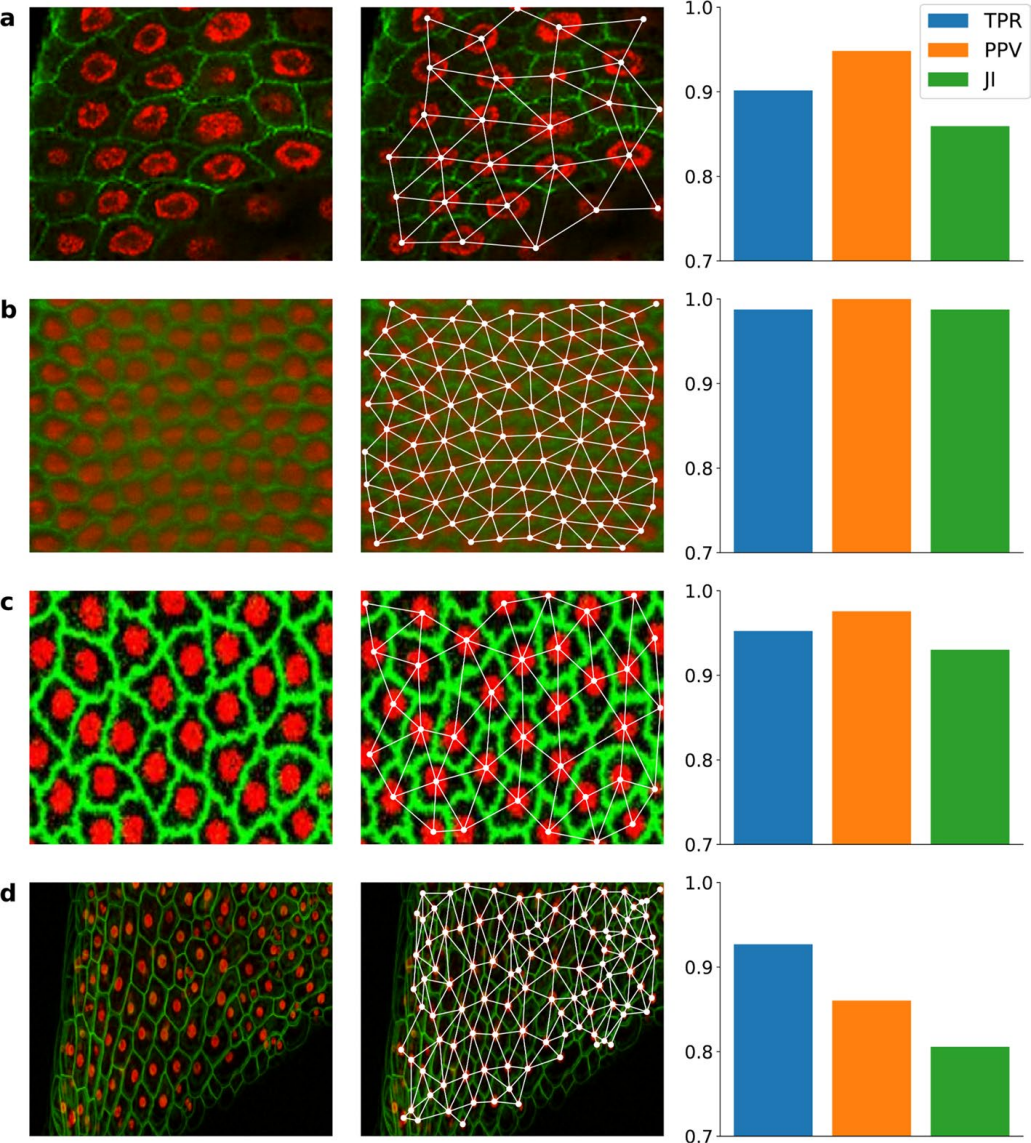
Neighboring cells estimation based on Nfinder. **a** Sample of intestinal epithelium from the adult midgut of *Drosophila melanogaster* immunostained for the cell periphery (green) and nucleus (red) (N = 27, TPR = 0.902, PPV = 0.948, JI = 0.859). Adapted from O’Brien and Bilder 2012 [24]. **b** *Drosophila* embryo imaged with SiMView microscopy. Nuclei are labelled with H2B-eGFP (red) and cell membranes are labelled with lyn-tdTomato under the control of the β-actin promoter (green) (N = 93, TPR = 0.988, PPV = 1.0, JI = 0.988). Adapted from Stegmaier et al*.* 2016 [25], **c** Average intensity projection of a uniform blastoderm of *Tribolium castaneum* embryo with H2B-RFP-labelled nuclei and GAP43-YFP-labelled membranes (N = 36, TPR = 0.952, PPV = 0.976, JI = 0.93). Adapted from Benton et al. [26]. **d** Confocal micrograph showing the expression of different fluorescent proteins in the stem of *Arabidopsis thaliana* (N = 105, TPR = 0.927, PPV = 0.86, JI = 0.806). Adapted from Federici and Haseloff [27]. First column: Original sample. Second column: filtered Delaunay triangulation of nuclei. Third column: performance metrics calculated by comparing the final result to the manually created ground truth. N, Number of cells; TPR, True Positive Rate; PPV, Positive predictive value. JI, Jaccard Index. Scale is not shown for images as the method is scale invariant

As a proof of concept, we applied Nfinder to different datasets available in the literature. Each dataset consisted of a single image that contained both nuclear and membrane fluorescent markers. The analyzed samples included (i) *Drosophila-1*: a sample of 27 intestinal epithelium cells from the adult midgut of *Drosophila melanogaster* immunostained for the cell periphery (bPS integrin) (Fig. 3a, green) and nucleus (DAPI) (Fig. 3a, red) [24], (ii) *Drosophila-2*: a Drosophila embryo containing 93 cells imaged with SiMView microscopy expressing H2B-eGFP to label the nuclei (Fig. 3b, red) and lyn-tdTomato under the control of the β-actin promoter to label cell membranes (Fig. 3b, green) [25], (iii) *Tribolium*: an average intensity projection of 36 cells from a uniform blastoderm of *Tribolium castaneum* embryo with H2B-RFP-labelled nuclei (Fig. 3c, red) and GAP43-YFP-labelled membranes (Fig. 3c, green) [26] and (iv) *Arabidopsis*: a confocal micrograph showing the expression of different fluorescent proteins in 105 cells from a stem of *Arabidopsis thaliana* (Fig. 3d) [27]. For details about each dataset, refer to Additional file 1: Fig. S1 (*Drosophila-1*), Additional file 1: Fig. S2 (*Drosophila-2*), Additional file 1: Fig. S3 (*Tribolium*) and Additional file 1: Fig. S4 (*Arabidopsis*).

The performance of Nfinder was tested by comparing our results to the manually generated ground truth for the neighboring cells graph of each dataset, as described in the previous section. For evaluation, we used metrics that do not depend on true negatives (TN). Such metrics were avoided because they are highly biased when negatives are dominant [28], which is the case given that there are many more possible links between cells (negatives) than links between cells that are actually touching (positives). In particular, we analyzed the True Positive Rate (TPR, also called sensitivity) (Eq. 4), Positive Predictive Value (PPV or precision) (Eq. 5) and Jaccard Index (JI) (Eq. 6).

After applying our automatic communicability efficiency filter, the percentage of links removed from the Delaunay triangulation was 20%, 11%, 12% and 13% for the datasets *Drosophila-1, Drosophila-2, Tribolium* and *Arabidopsis*, respectively. As it is shown in Fig. 3, TPR was over 90% in all cases, supporting the idea that the Delaunay triangulation of nuclei provides a good estimation of neighboring cells. In addition, PPV was 94.6% on average and a maximum of 100% was reached in a *Drosophila* embryo tissue with highly regular spatial distribution (Fig. 3b). This indicates that the more regular the tissue, the better the method’s performance. However, its range of application has proven wider as results were satisfactory in irregular tissues, such as the one from the *Arabidopsis* dataset (Fig. 3d). Even in this case, the poorest performance was 80% in JI.

### Nfinder is easily extensible to 3D datasets

**Fig. 4:**
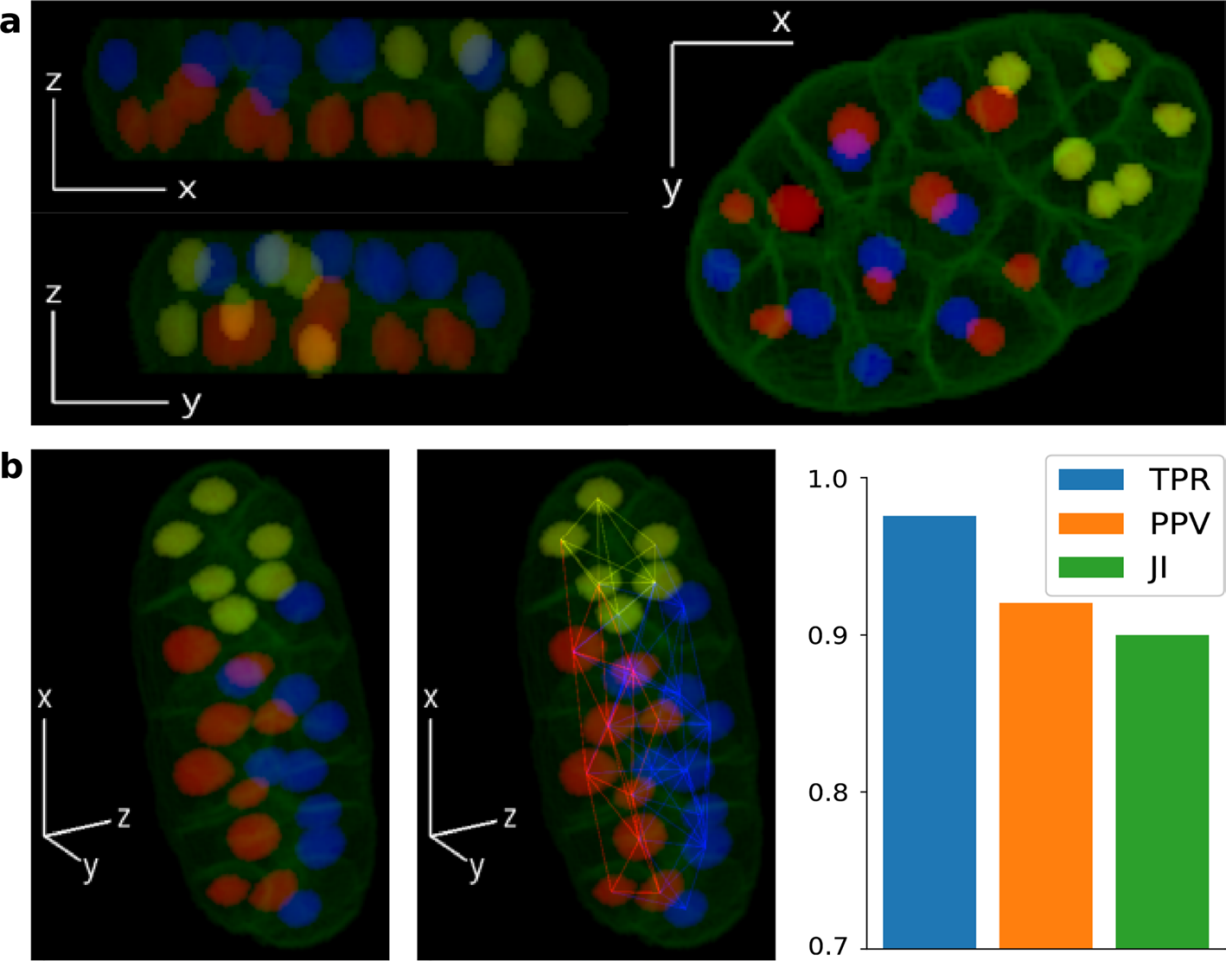
Nfinder results in 3D. **a** Planar views of a 3D stack of a *C. elegans* embryo, showing cell membranes (green) and nuclei (N = 24). Colors were chosen so that the embryo was divided by a plane in $z =$ 8.83 μm (red: $z <$ 8.83 μm; blue: $z \ge$ 8.83 μm). Detected neighbors of the cell with the maximum x-position are highlighted (yellow). Adapted from Azuma and Onami 2017 [29]. **b** Final results: 3D view of the analyzed specimen (left), estimated cell graph (center) and performance metrics (right; TPR = 0.976, PPV = 0.92, JI = 0.9). N, Number of cells; TPR, True Positive Rate; PPV, Positive Predictive Value; JI, Jaccard Index. Scale is not shown for images as the method is scale invariant

As Delaunay triangulations exist in all n-dimensional spaces, Nfinder can be easily extended to 3D stacks. In contrast to the planar space, the basic structure of the 3D Delaunay network is not a triangle but a tetrahedron. Therefore, before applying the proposed method, each tetrahedron is decomposed into four triangles. Next steps of the pipeline are the same as those described in the previous sections. To show the usefulness of Nfinder in 3D, we estimated the neighboring cells graph of a 3D stack of a *Caenorhabditis elegans* embryo with 24 cells (Fig. 4a and Additional file 2: Video S1), whose nuclei and membranes were stained with mCherry and GFP, respectively [29]. To facilitate the visualization of overlapping cells, we color-coded nuclei according to their position in *z*, and highlighted the nucleus with the maximum x-coordinate and its neighbors.

In this case, the percentage of links removed from the Delaunay triangulation by automatic communicability efficiency filtering was 16%. As shown in Fig. 4b, our method was successful in all performance metrics ($\ge 90\%$), with a remarkable TPR of 97.6%.

## Discussion

We developed Nfinder, an automatic and robust method for estimating neighboring relationships between cells using only the information provided by nuclear markers. This is a remarkable feature because membrane staining is not always possible nor convenient in many applications, ranging from cell signalling to tissue development. The robustness of our method is mostly determined by making an educated guess of neighbor relationships between cells via a Delaunay triangulation, which makes results stable against small displacements of cells. In particular, this property is useful in cell tracking, as it is expected that small fluctuations in the positions of cells between timepoints do not affect their connection. Regardless of being designed for nuclear markers, Nfinder can also be applied to cell centroids whenever cytosolic or cytoplasmic fluorescent markers are available.

Although similar strategies have been used before [15, 30, 31], previous works were not focused on the detection of cell neighbors and, therefore, their performance was not thoroughly analyzed. In addition, links between cells were only filtered by a distance threshold, neglecting the importance of non-pairwise interactions [31]. Furthermore, this threshold was an input of the algorithm and, thus, its reusability and generalization were user-dependent. Nfinder is the first method to infer neighboring cells from nuclei labelling that is (i) fully automatic and (ii) includes non-pairwise interactions. The automation of our method was achieved by maximizing a new empirical statistic that assigns a communicability efficiency to each edge of the Delaunay triangulation (Additional file 1: Fig. S5). As for the quantification of non-pairwise interactions, we used the communicability angle of each pair of cells, defined as the maximum angle subtended with shared neighbors.

The proposed automatic filtering of the Delaunay triangulation based on the communicability efficiency of the cell graph improved the performance of the neighboring estimation. To test this, we compared the TPR, PPV and JI obtained for all datasets by selecting edges based on distance thresholding to our method’s results (Additional file 1: Fig. S6). This analysis was performed following the protocol explained in the Methods section. Here, the distance threshold was chosen by maximizing the communicability efficiency modeled as

9 $$E\left( r \right) = F\left( r \right) - L\left( r \right)$$

where the cumulative distribution of edges $F$ and the loss function $L$ only depend on the distance threshold $r$. Here, $L$ was formulated as

10 $$L\left( r \right) = \frac{{r - r_{min} }}{{r_{ma x} - r_{min} }}$$

being $r_{min}$ and $r_{max}$ the minimum and maximum edge length in the Delaunay triangulation, respectively. Considering $r$ as the distance threshold, this loss function represents the fraction of edges that would be filtered out from the Delaunay triangulation if the distance between cells followed a uniform distribution between $r_{min}$ and $r_{max}$. Contrasting the performance metrics of the described distance thresholding with our method’s results, we observed that, on average, incorporating the communicability angle increases the JI by + 4.2% and PPV by + 5.6%, while the TPR did not change significantly (-0.1%). Remarkably, this contribution improved PPV by + 11.5% in the dataset *Drosophila-1*. Performance differences seem to be more noticeable when the image contains a low number of cells (*Drosophila-1*) or the spatial distribution of cells is more complex (*C. elegans*). These aspects could be relevant, for instance, when tracking cell–cell interactions during the early stages of embryonic development in 3D.

Importantly, we observed that the performance of Nfinder is affected by how regular the geometry of the cell graph is. This may be due to the correspondence between the Delaunay triangulation and the Voronoi diagram. Since Voronoi cells are convex polygons, neighboring relations between irregular-shaped cells, such as dendritic cells or neurons, might not be well approximated by the Delaunay triangulation of nuclei centroids. A solution to this problem could be replacing these with a more representative set of points, determined by non-convex structures [32]. This analysis is beyond the scope of this work and could be the purpose of future research. However, the validation of our method showed that it is highly accurate and versatile, working with similar performance in diverse tissues both in 2D and 3D.

## Conclusion

In this paper, we developed Nfinder, a precise and automatic method to find neighboring cells from nuclei labelling in 2D and 3D. This automation was carried out by maximizing a novel statistic that measures the communicability efficiency of a spatial graph. Unlike previous works, our method includes cell–cell and non-pairwise interactions, which improves the detection performance significantly. After a systematic evaluation of our method’s performance, we found that the importance of considering non-pairwise interactions cannot be neglected if the number of cells is low or the cell spatial distribution is non-uniform. We believe that using our approach might add biologically relevant information to other pipelines, especially those aiming at tracking cell–cell interactions and studying spatiotemporal correlations at the tissue level.

## Supplementary Information

**Additional file 1.** Supplementary Figures S1–S6. Additional performance metrics.

**Additional file 2. Video S1:** 3D animation of *C. elegans* embryo showing the estimated neighboring cell graph.

## Data Availability

Image datasets used in this manuscript can be found in references [24–27]. Project name: Nfinder. Project home page: https://github.com/santi-rodriguez/nfinder. Operating system(s): Platform independent. Programming language: Python. Other requirements: Python 3.7 or higher, License: MIT License. Any restrictions to use by non-academics: None.

## Abbreviations

CIL: Contact inhibition of locomotion
ECDF: Empirical Cumulative Distribution Function
TP: True positive
FP: False positive
FN: False negative
TN: True negative
TPR: True Positive Rate
PPV: Positive Predictive Value
JI: Jaccard Index
GFP: Green fluorescent protein
