# Supplementary material for: Nfinder: automatic inference of cell neighborhood in 2D and 3D using nuclear markers
Source: BMC Bioinformatics. 2023 Jun 3;24:230. doi: 10.1186/s12859-023-05284-2 (PMC10239575; doi:10.1186/s12859-023-05284-2)
Supplement: Supplementary file 1 — Additional file 1. Supplementary Figures S1–S6. Additional performance metrics. [file 12859_2023_5284_MOESM1_ESM.docx]

**Automatic inference of cell neighborhood in 2D and 3D using nuclear markers**

**Bruno Moretti,^a,b,1,†,*^ Santiago N. Rodriguez Alvarez^a,1, †^, Hernán E. Grecco^a,b,*^**

^a^ Department of Physics, FCEN, University of Buenos Aires, Buenos Aires, Argentina

^b^ Instituto de Física de Buenos Aires, CONICET, Buenos Aires, Argentina

^1^ These authors contributed equally to this work.

† Current address: B.M.: Department of Molecular and Cell Biology, University of California at Berkeley

S.N.R.A: Institute of Physics, Swiss Federal Institute of Technology Lausanne (EPFL), Lausanne, Switzerland

*****Corresponding authors**:** Bruno Moretti ([morettibruno@gmail.com](mailto:morettibruno@gmail.com)) and Hernán E. Grecco ([hgrecco@df.uba.ar](mailto:hgrecco@df.uba.ar))

**Appendix A: Supplemental Material**

**
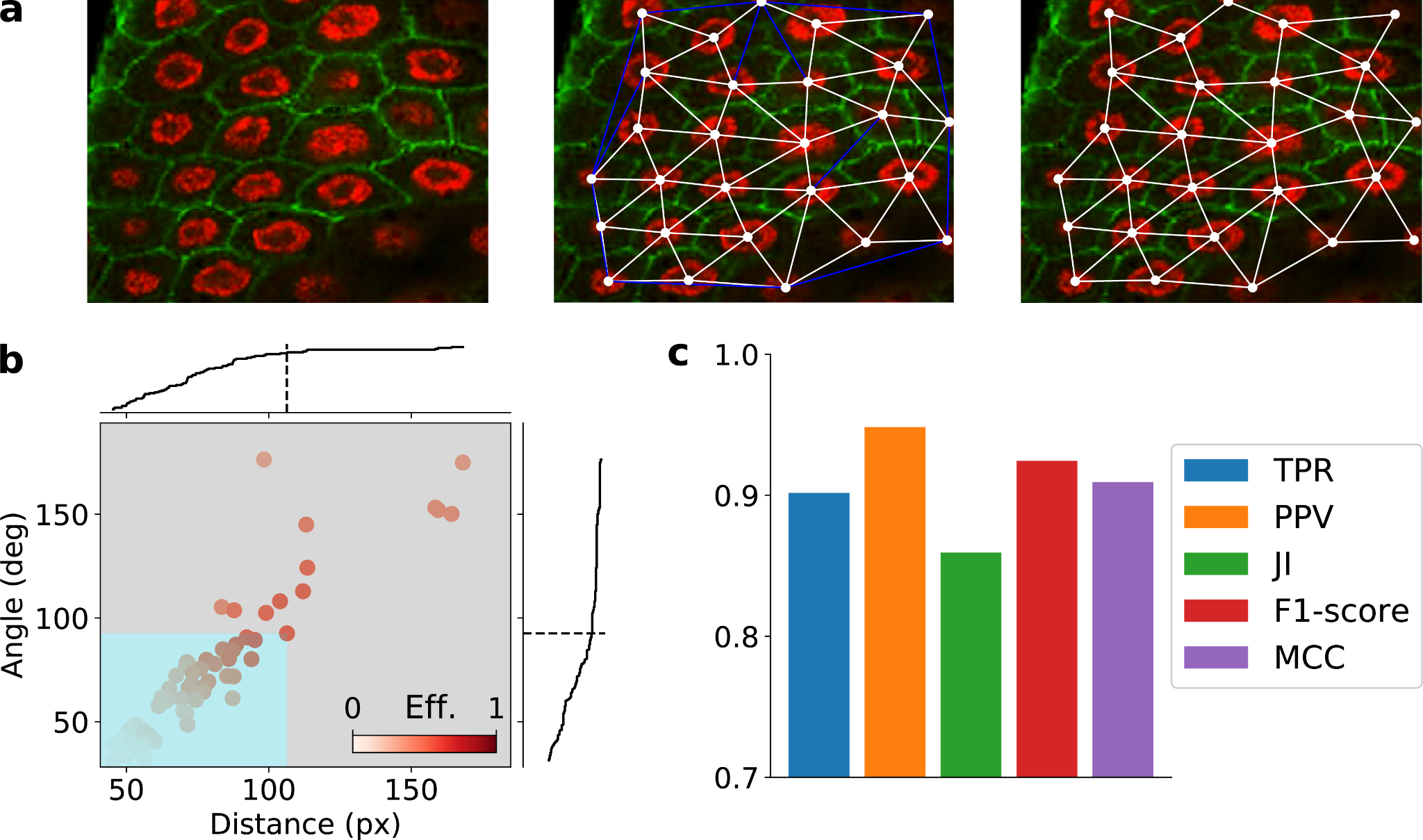
Fig. S1** Results obtained for the dataset *Drosophila-1.* **(a)** Delaunay triangulation of nuclei centroids and edge filtering by communicability efficiency. **Left**: Original sample. **Center**: Delaunay triangulation, including errors (blue). **Right:** filtered neighboring cell graph. **(b)** Distribution of Delaunay edges in the distance-angle plane. Each point represents a unique edge in the Delaunay triangulation and is colored by communicability efficiency. Coordinates of the point with maximum communicability efficiency correspond to the threshold values of distance and angle, which divide the plane in positives (cyan) and negatives (gray). **(c)** Performance metrics calculated by comparing the estimated graph to the manual ground truth. **TPR**: True Positive Rate. **PPV**: Positive Predictive Value. **JI**: Jaccard Index. **MCC:** Matthews correlation coefficient. Scale is not shown for images as the method is scale invariant.

**
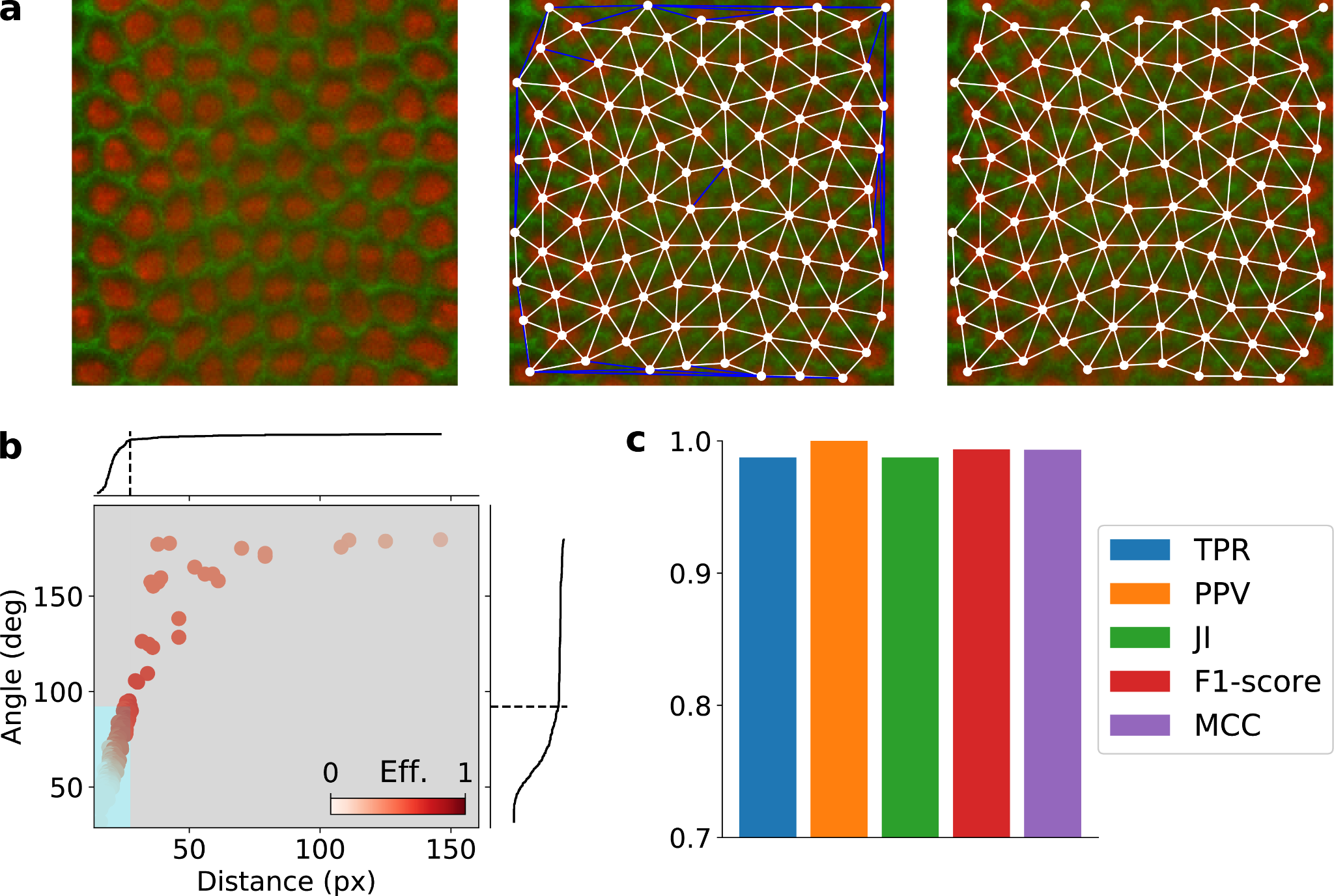
**

**Fig. S2** Results obtained for the dataset *Drosophila-2.* **(a)** Delaunay triangulation of nuclei centroids and edge filtering by communicability efficiency. **Left**: Original sample. **Center**: Delaunay triangulation, including errors (blue). **Right:** filtered neighboring cell graph. **(b)** Distribution of Delaunay edges in the distance-angle plane. Each point represents a unique edge in the Delaunay triangulation and is colored by communicability efficiency. Coordinates of the point with maximum communicability efficiency correspond to the threshold values of distance and angle, which divide the plane in positives (cyan) and negatives (gray). **(c)** Performance metrics calculated by comparing the estimated graph to the manual ground truth. **TPR**: True Positive Rate. **PPV**: Positive Predictive Value. **JI**: Jaccard Index. **MCC:** Matthews correlation coefficient. Scale is not shown for images as the method is scale invariant.

**
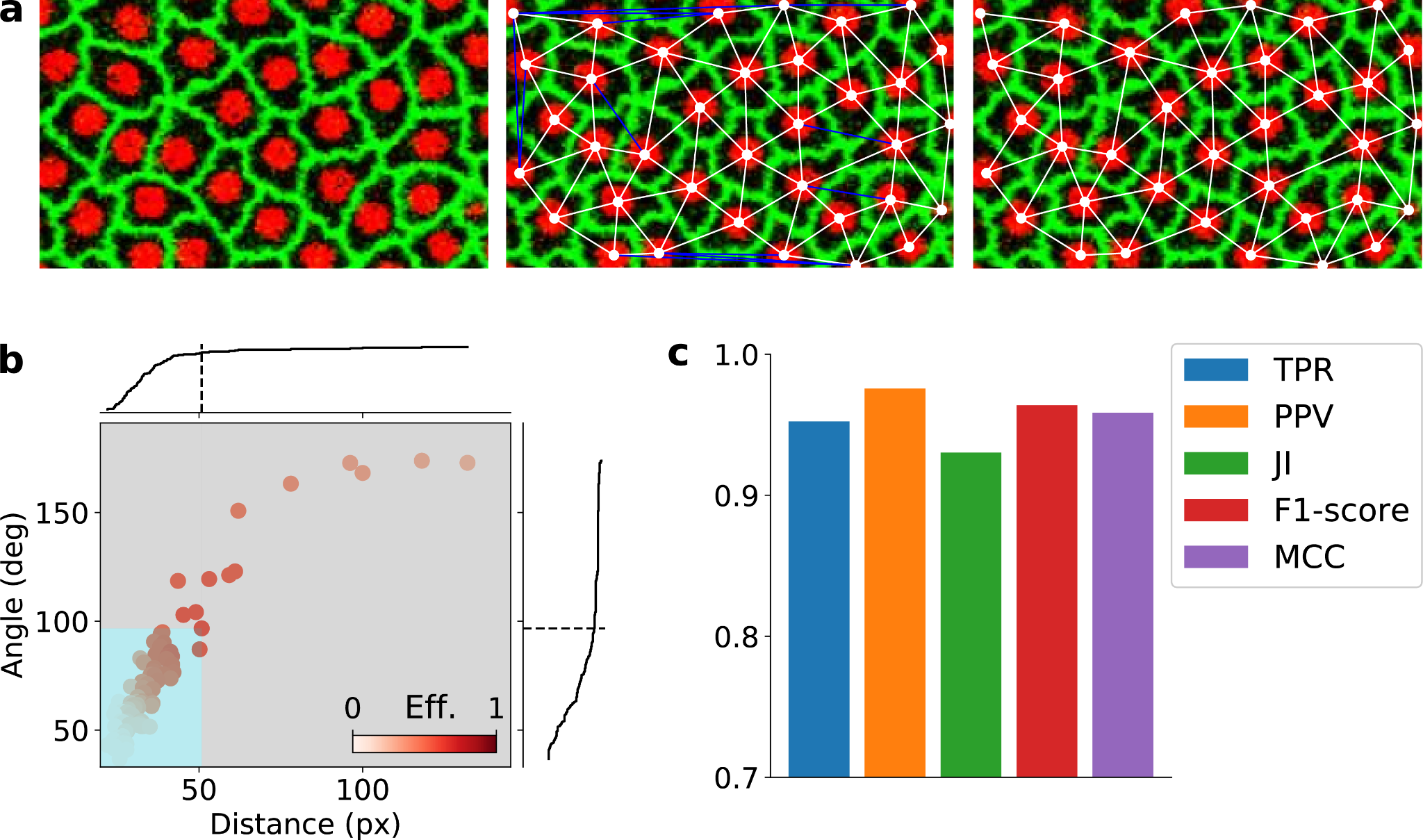
**

**Fig. S3** Results obtained for the dataset *Tribolium.* **(a)** Delaunay triangulation of nuclei centroids and edge filtering by communicability efficiency. **Left**: Original sample. **Center**: Delaunay triangulation, including errors (blue). **Right:** filtered neighboring cell graph. **(b)** Distribution of Delaunay edges in the distance-angle plane. Each point represents a unique edge in the Delaunay triangulation and is colored by communicability efficiency. Coordinates of the point with maximum communicability efficiency correspond to the threshold values of distance and angle, which divide the plane in positives (cyan) and negatives (gray). **(c)** Performance metrics calculated by comparing the estimated graph to the manual ground truth. **TPR**: True Positive Rate. **PPV**: Positive Predictive Value. **JI**: Jaccard Index. **MCC:** Matthews correlation coefficient. Scale is not shown for images as the method is scale invariant.

**
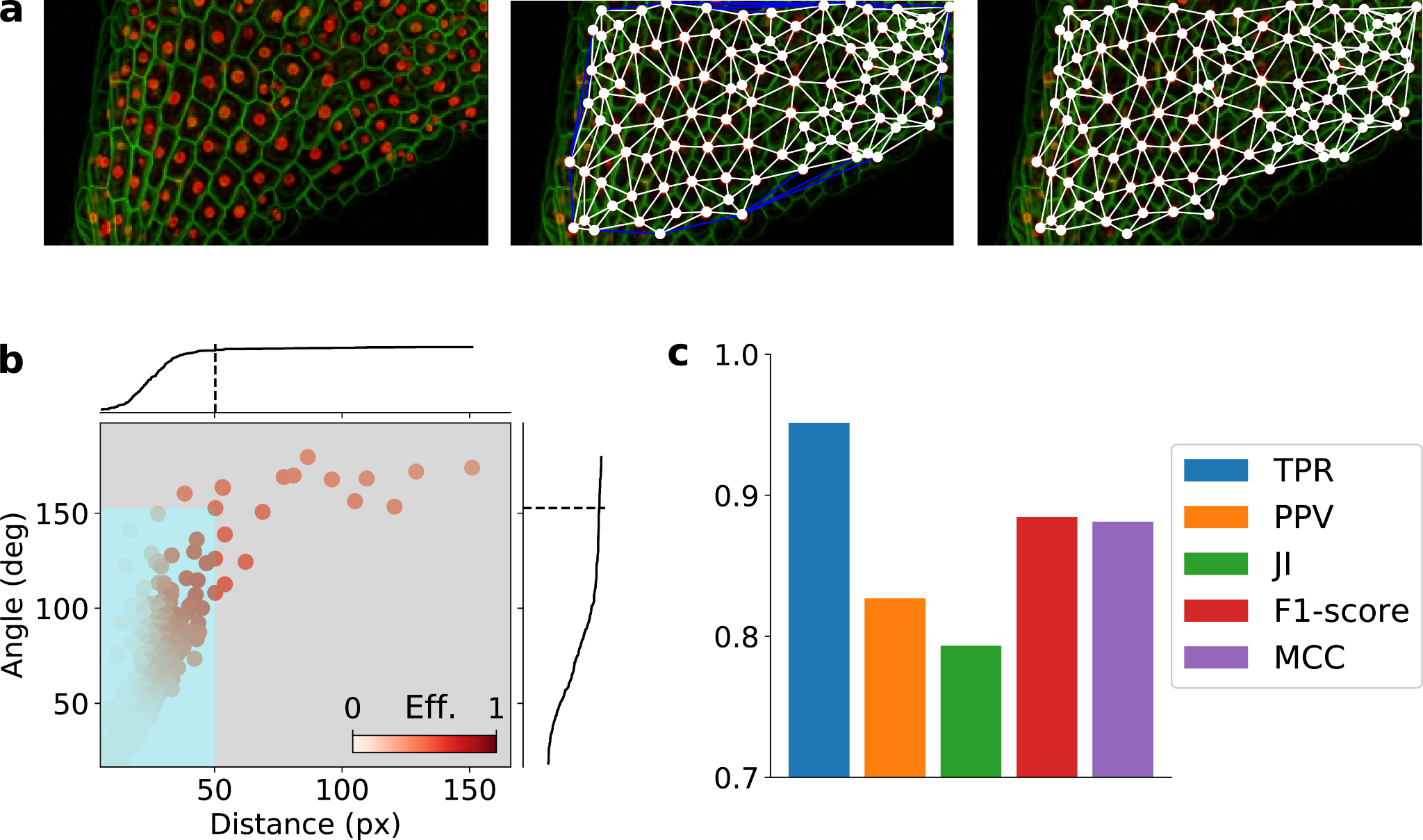
**

**Fig. S4** Results obtained for the dataset *Arabidopsis*. **(a)** Delaunay triangulation of nuclei centroids and edge filtering by communicability efficiency. **Left**: Original sample. **Center**: Delaunay triangulation, including errors (blue). **Right:** filtered neighboring cell graph. **(b)** Distribution of Delaunay edges in the distance-angle plane. Each point represents a unique edge in the Delaunay triangulation and is colored by communicability efficiency. Coordinates of the point with maximum communicability efficiency correspond to the threshold values of distance and angle, which divide the plane in positives (cyan) and negatives (gray). **(c)** Performance metrics calculated by comparing the estimated graph to the manual ground truth. **TPR**: True Positive Rate. **PPV**: Positive Predictive Value. **JI**: Jaccard Index. **MCC:** Matthews correlation coefficient. Scale is not shown for images as the method is scale invariant.


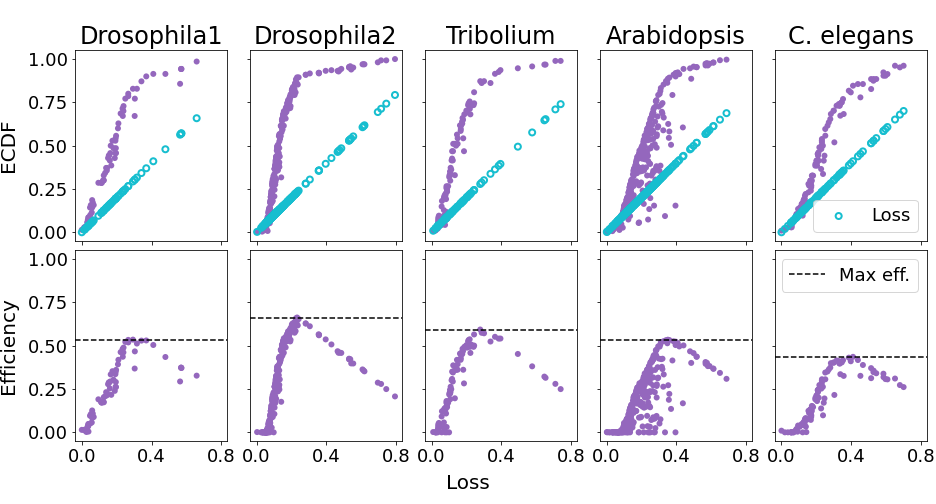


**Fig. S5** Estimation of communicability efficiency for all datasets. Points correspond to edges in the Delaunay triangulation. **First row:** Empirical Cumulative Distribution Function (ECDF, *F* in Eq. 1) (purple) versus mean loss. To provide a reference, the mean loss function (identity, *L* in Eq. 2) (cyan) is shown. **Second row:** Communicability efficiency as a function of the mean loss (purple). This curve is obtained by subtracting the mean loss to the ECDF. Negative values were mapped to zero. For each dataset, the maximum communicability efficiency is highlighted (dashed line).


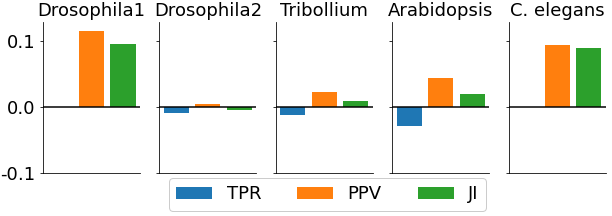


**Fig. S6** Performance metrics changes between the proposed method and a cell-cell distance filter. The figure shows the difference of the TPR, PPV and JI between Nfinder and a method that approximates the cell graph as the Delaunay triangulation filtered by cell-cell distance thresholding. The comparison suggests that filtering edges not only by distance but also by communicability angle improves the approximation of the cell graph, especially when the number of cells is low(*Drosophila-1*) or the spatial distribution of cells is complex (*C. elegans*). The maximum difference corresponds to the *Drosophila-1* dataset*,* where our method has a PPV advantage of +11.5%, compared to distance-only thresholding. **TPR**: True Positive Rate. **PPV**: Positive Predictive Value. **JI**: Jaccard Index.
